# Words to Young Homœopaths

**Published:** 1887-06

**Authors:** D. C. McLaren

**Affiliations:** Brantford, Ontario


### WORDS TO YOUNG HOMŒOPATHS.

D. C. McLaren, B. A., M. D., Brantford, Ontario.

(Concluded from April Number).

Though I had several times read the *Organon*, it was not till recently that I made a searching study of that intricate work; the more I read and study it, the more I am amazed at the wealth of observation, the profound discrimination, and logical deduction displayed by Hahnemann. A history of his life would furnish the clue to his remarkable success. For many years—at least twelve, probably twenty—the days were not sufficient for the engrossing work he was engaged in, so he crowded time by laboring and sleeping alternate nights. Such indefatigable perseverance always brings its reward, and will do so for young homœopaths to-day, as it did for Hahnemann. To him was reserved the supreme dignity of having first and alone discovered the cause and cure of chronic diseases. Hear what he says (note, § 80.)

"Until I had examined the depths of this important matter, it was impossible for me to teach the mode of subduing all chronic diseases except as isolated and individual affections by the medicinal substances that were till then known according to their effects upon healthy persons, so that the followers of my method treated each case of chronic disease separately as a distinct group of symptoms, which, however, did not prevent their cure to such an extent that suffering humanity had good cause to rejoice at the newly discovered system of medicine."

But before entering on the subject of *Chronic Diseases*, Hahnemann gives the definition of chronic diseases proper and of those improperly so called—the latter first, as follows: "§ 77. The name chronic is very improperly applied to those diseases which attack persons who are constantly exposed to baleful influences from which they might have screened themselves—persons who constantly make use of aliments or drink that are hurtful to the system; who commit excesses that are injurious to health; who are every moment in want of the articles necessary to support life, and who inhabit unwholesome countries, and, above all, marshy places; who live in cellars and other confined dwellings, who are deprived of air and exercise, who are exhausted by immoderate labor of the mind or body; who are consumed by perpetual ennui, etc. These diseases, or, rather, these privations of health, brought on by individuals, disappear of themselves by a mere change of regimen, provided there is no chronic miasm in the body, but they cannot be called chronic diseases."

Hahnemann says, in the next section, that "the true natural chronic diseases are those which are produced by a chronic miasm, making continual progress in the body when no specific curative remedy is opposed to them, and which, notwithstanding all imaginable care, both with regard to the regimen of the body and mind, never cease tormenting the patient with an accumulation of miseries that endure till the latest period of his existence."

These chronic miasms are three in number—syphilis, sycosis, and psora, the last-named being Hahnemann's much derided, but, for all that, great and sublime discovery. To those who deem it absurd, and to those who have never given it a thought, I would say, prove it for yourselves. But how? Well, you have not been long in practice before you encounter chronic ailments, which you undertake to cure hopefully and expectantly. The first prescription, and possibly the second, gives good results if symptoms have been well covered, but after that no amount of individualizing and symptom covering do more than apparently aggravate the case. The writer had a very instructive case, the treatment of which extended over five years. The lady came first from allopathic hands; she had been failing rapidly, and my diagnosis was incipient phthisis, though I could not locate any tuberculous lesion; in addition, she had retroversion of the uterus, ulceration of the os, obstinate constipation, protruding piles, and excessive weakness and pain of the back in consequence. *Æsculus* was the first remedy; it gave some relief to the back and hemorrhoids, but alarming oppression of the chest soon required *Phosphorus*, and before long the uterus demanded attention and got *Pulsatilla.* For some months then there was an alternation of *Hepar* and *Pulsatilla,* as either chest or uterus troubled her most, the doses being generally some weeks apart. Finally I consulted Dr. Ad. Lippe, who recommended *Sulphur,* though, as far as I could discover, it had but few of her symptoms. For two or three years she got a dose of Sulphur about once a month, bore a healthy child in the interval, and during the last year of treatment got a dose or two of Calcarea. She has not required a prescription of any kind for upward of a year. What I learned from that case might and should have been taught me in college, but failing that, our journals must take up the *role* of teachers.

In all chronic cases and in most of those described by Hahnemann as privations of health, wherein the detrimental habit of life has had sufficient scope to work upon and generate from the latent psora definite disease types, the elimination of this psoric factor must be the first object of the earnest homœopath. For instance, if the symptoms seem to demand *Belladonna* in a case of long standing, the proper antipsoric required to effect a cure will probably be *Calcarea,* and in some cases *Lycopodium,* according as the symptoms direct. Again, the acute manifestations of a chronic ailment demand *Nux. Sulphur* must subsequently be given to effect a cure. So, too, may be paired, Aconite and Sulphur, Allium cepa and Calcarea, Æthusa and Calcarea, etc. Of course, these are not hard-and-fast rules. Often several antipsorics may be needed, and sometimes unusual ones, but a clear understanding of the principle will lead to a proper and successful application of the remedies, and without it—well, their works are known!

But, says one, all this does not prove the truth of the psora theory. Possibly not; but it does prove, emphatically, the existence of a single chronic miasm, else how can so many diverse forms of disease be cured by the same remedies? As to *naming* that chronic miasm, I am perfectly willing to follow Hahnemann, and call it psora, the many important cures made by the nosode *Psorinum* being to me sufficient evidence on that point.

It is highly important in both acute and chronic ailments to discover as part of the diagnosis, prognosis, and treatment whether the case in point be what Hahnemann calls a "privation of health" or a distinct malady arising from a chronic miasm, and if the latter, to what extent it is influenced, obscured, and rendered obstinate by faulty habits of living.

A case or two will illustrate conclusively. A near relative, whose chronic headaches I have had an opportunity of treating for some years, but always unsuccessfully, was induced a few months since to part with his only stimulant, *tea*, which for many years he drank three times a day. As a result, his general health has greatly improved, and not a single headache has he had since. He has taken quite a dislike to the tea, and considers himself cured of the old trouble. Many apparently incurable disorders can be traced indubitably to the use of tea, coffee, tobacco, and stimulants.

But on this point just one word—it is seldom wise to tell a man that tobacco is hurting him. He will simply call in another physician. Use antidotes, and patch up the case the best way you can, and let him pay for his indulgence by consulting you from time to time.

Dr. H. C. Allen relates a case of strangury and retention formerly treated by the allopaths mechanically, but promptly relieved by the Doctor with *Opium*. The man being a smoker, the allied narcotic was both antidotal and symptomatic. Several attacks were cured, when finally the patient insisted on knowing the cause of the disorder and got the desired information, but the Doctor lost a patient! Most men (and women, too) will go pretty close to death's door before giving up their favorite habit!

